# Supplementary material for: Early childhood investment impacts social decision-making four decades later
Source: Nat Commun. 2018 Nov 20;9:4705. doi: 10.1038/s41467-018-07138-5 (PMC6246600; doi:10.1038/s41467-018-07138-5)
Supplement: Supplementary file 1 — Supplementary Information [file 41467_2018_7138_MOESM1_ESM.pdf]

Supplementary Information for

Early childhood investment impacts social decision-making four decades later

Luo et al.

This file includes:

Supplementary Figure 1-5

Supplementary Table 1-5

Supplementary Notes 1-6

Supplementary References

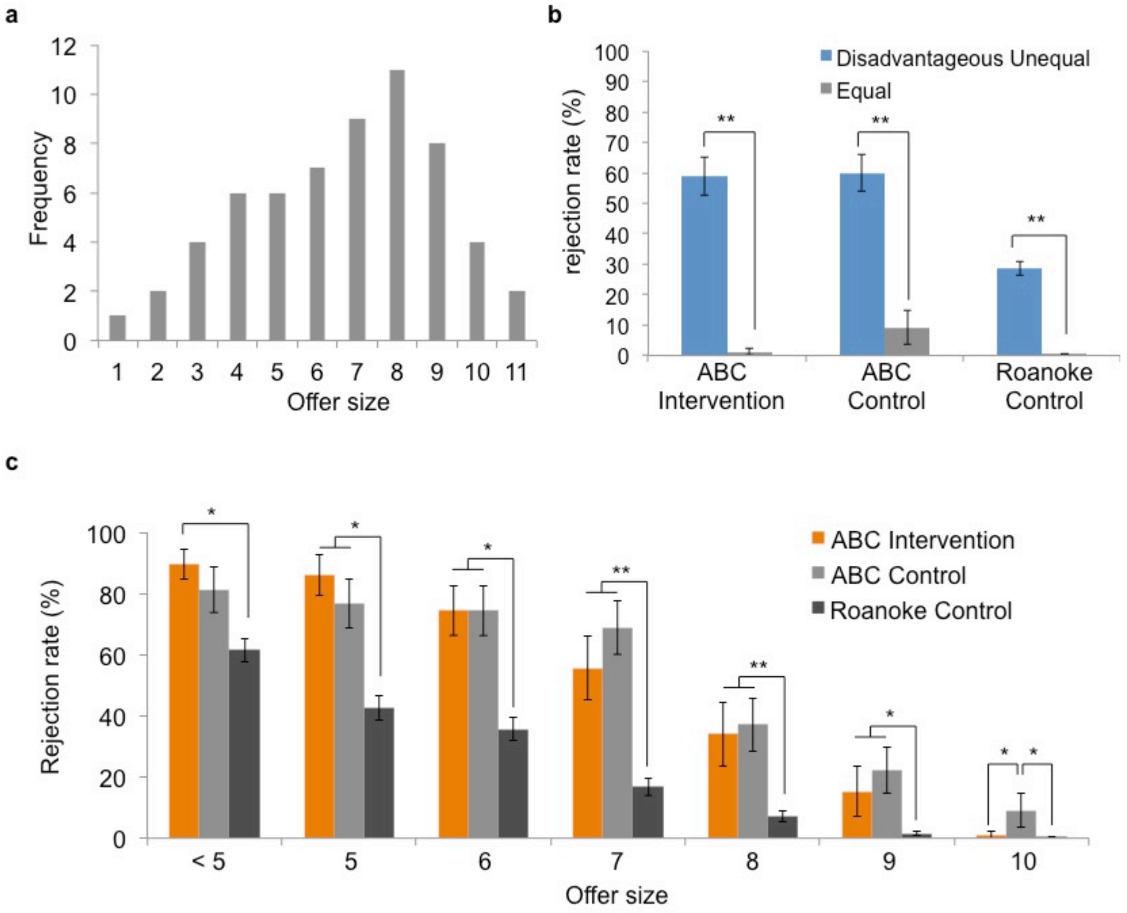

Supplementary Figure 1. Offer distribution and rejection rates across 60 rounds in Medium-Low-Medium (MLM) type. (**a**) Distribution of offer size for MLM. The frequency is the average occurrence for each offer size across participants in MLM. (**b**) Rejection rates for MLM grouped by level of equality. All groups rejected disadvantageous offers more than equal offers. However, Roanoke Controls rejected disadvantageous offers less than ABC Interventions and ABC Controls, while ABC Controls rejected equal offers more than ABC Interventions and Roanoke Controls. (**c**) Rejection rates for MLM grouped by offer size. Roanoke Controls rejected offers at most of the disadvantageous offers less than ABC Interventions and ABC Controls except for offers < 5. However, ABC Controls rejected equal offers more than ABC Interventions and Roanoke Controls. * $p < .05$, ** $p < .001$ (post hoc t test). Error bar represents s.e.m.

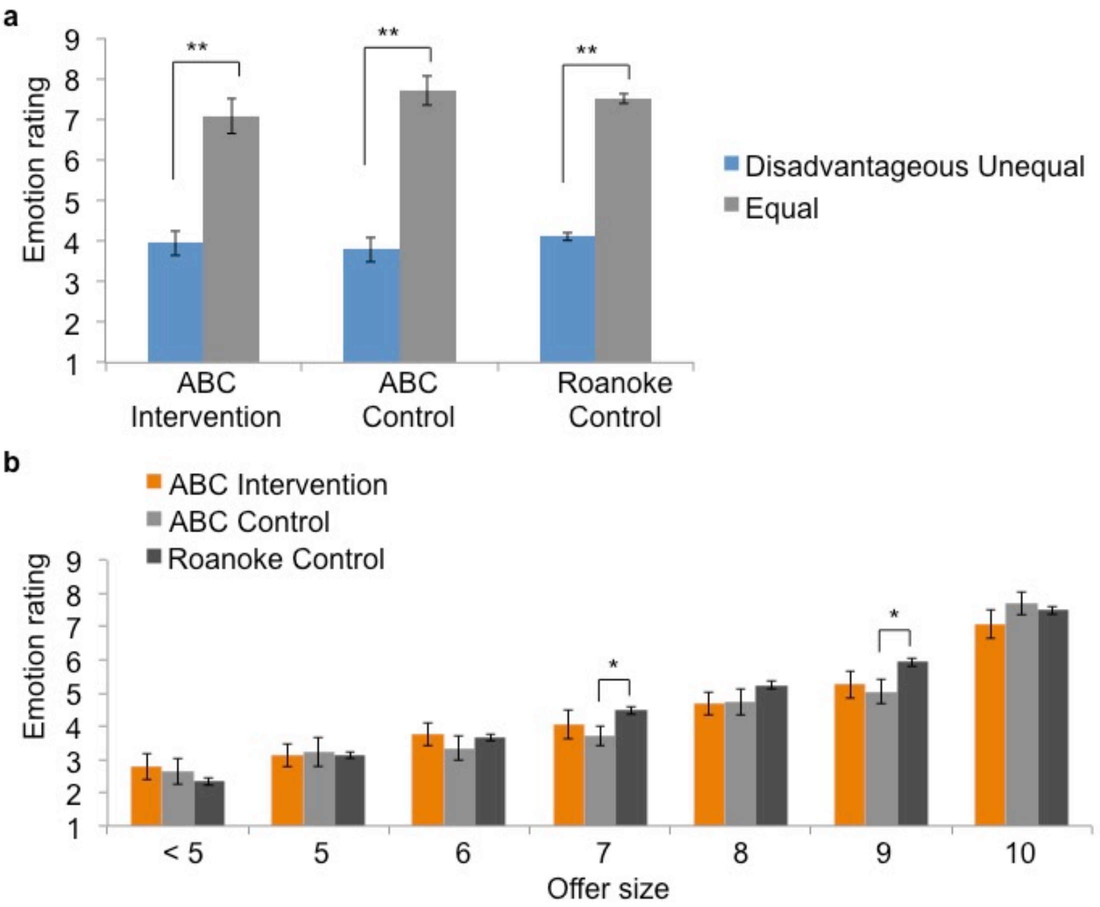

Supplementary Figure 2. Emotion rating across 60 rounds in Medium-Low-Medium (MLM) type. (**a**) Emotion rating for MLM type grouped by level of equality. No Group (ABC Intervention vs. ABC Control vs. Roanoke Control) × Equality (Disadvantageous Unequal vs. Equal) interaction in MLM was found. Disadvantageous unequal offers were rated as less pleasant than equal offers. No difference on emotion rating was found among the three groups. (**b**) Emotion rating across 60 rounds in MLM type grouped by offer size. A Group × Offer Size interaction in MLM was significant, with Roanoke Controls only rating offers = 7 and offers = 9 as more pleasant than ABC Controls. * $p < .05$, ** $p < .001$ (post hoc *t* test). Error bar represents the s.e.m.

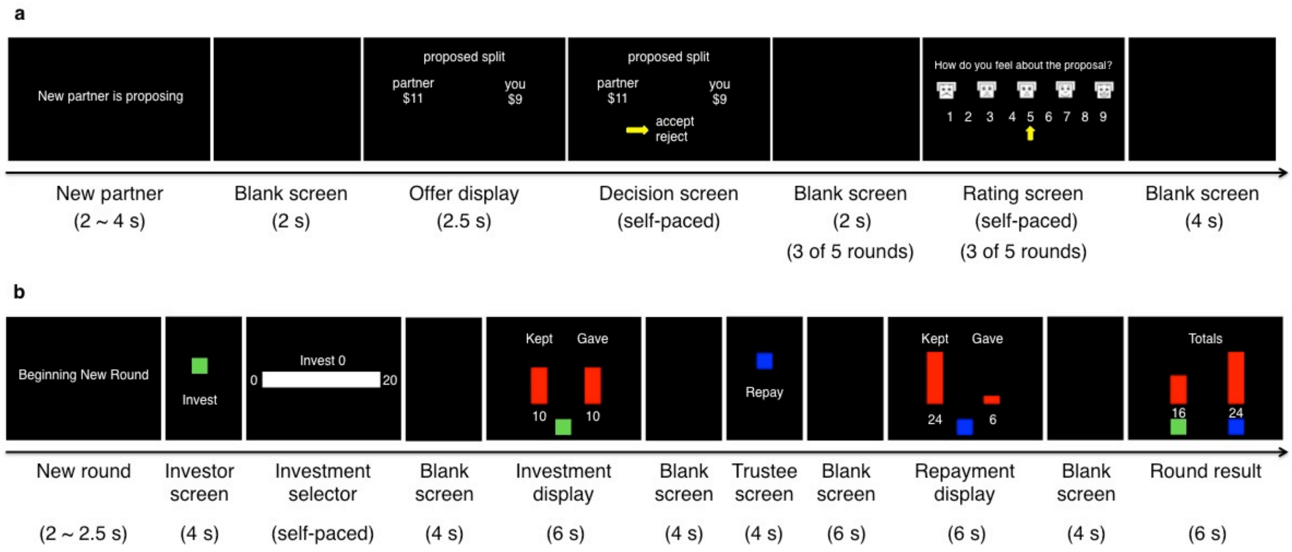

Supplementary Figure 3. (**a**) Procedure of a single round in the ultimatum game. Participants playing as the Responder were told that they were paired with a new Proposer, then received the offer, chose to accept or reject, and finally, on three out of five rounds, were asked to rate their feelings about the current offer on a 1-9 scale. (**b**) Procedure of a single round in the multi-round trust game. Participants were told that they were the Investor who could choose any portion of $20 to send to the trustee who then decide how much of the tripled investment to repay, followed by the outcome of the current round.

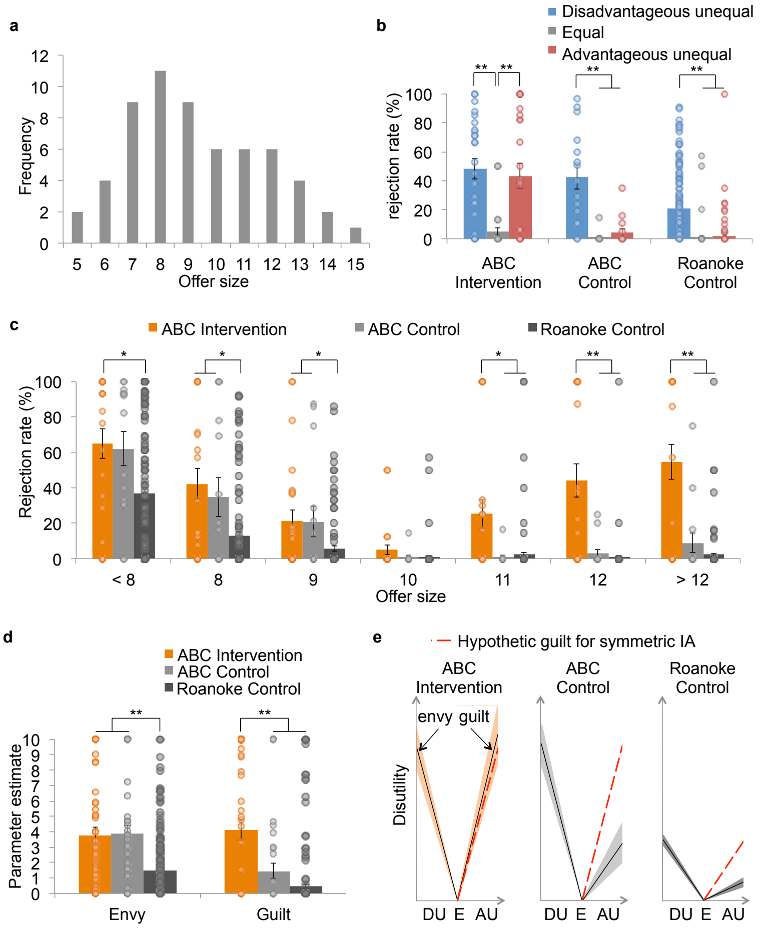

Supplementary Figure 4. Offer distribution, rejection rates and model based parameters in the ultimatum game. (**a**) Distribution of offer size for Medium-High-Medium (MHM) conditioning type. The frequency is the average occurrence for each offer size across participants in MHM. (**b**) Rejection rates for MHM grouped by level of equality. All groups had higher rejection rates for disadvantageous offers than equal offers. Advantageous offers were not rejected more than equal offers in ABC Control and Roanoke Control, while ABC Interventions rejected advantageous offers more than equal offers. (**c**) Rejection rates for MHM grouped by offer size. Only ABC Interventions increased rejection rates as a function of inequality, regardless of them being personally advantageous or disadvantageous, presenting a "V shape" pattern. (**d**) Parameter estimates from the behavioral modeling using a Fehr-Schmidt inequality aversion model. Both ABC Interventions and ABC Controls have a higher level of envy (unwillingness to accept unequal offers which are disadvantageous to the participant) than Roanoke Controls. The ABC Interventions had a higher guilt (unwillingness to accept unequal offers which are advantageous to the participant) than ABC Controls and Roanoke Controls. (**e**) The horizontal axis presents different level of equality: disadvantageous unequal (DU); equal (E); advantageous unequal (AU)). The vertical axis presents the disutility defined by the inequality aversion (IA) model (i.e., sensitivity × inequality). The slope of each line presents the sensitivity for inequality aversion (IA; envy for DU and guilt for AU). A steeper slope corresponds to higher inequality aversion. Compared with the control groups, ABC Interventions presented a much more symmetric IA pattern (i.e., the same level of envy and guilt). Every dot represents rejection rate (in **b** and **c**) or parametric estimate (in **d**) for one individual participant in the corresponding condition. Shaded areas are bounded by mean ± s.e.m. * $p < 0.05$, ** $p < 0.001$(post hoc t test p-values). Error bar represents s.e.m.

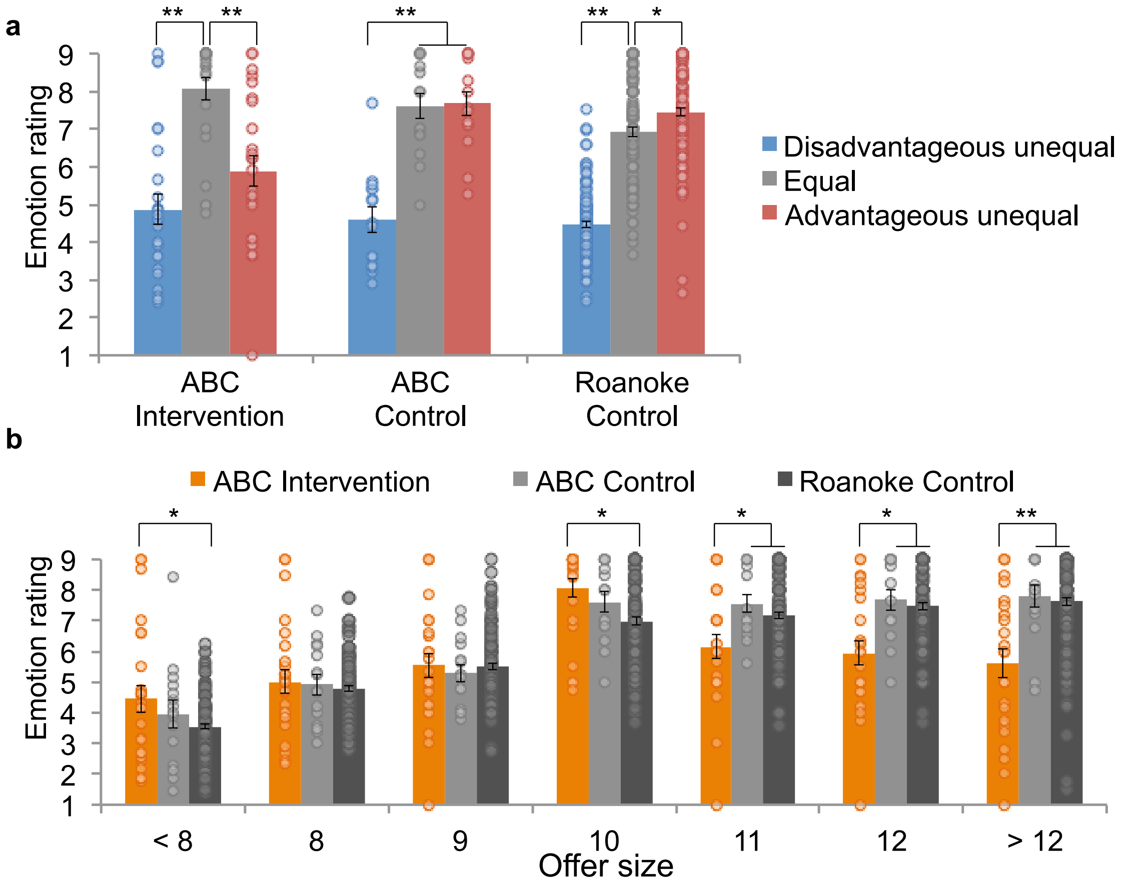

Supplementary Figure 5. Emotion rating across 60 rounds in Medium-High-Medium (MHM) type in the ultimatum game. (**a**) Emotion rating for MHM grouped by level of equality. ABC Interventions rated equal offers as more pleasant than both disadvantageous and advantageous offers. ABC Controls rated disadvantageous offers as less pleasant than equal offers but did not report different feelings about equal and advantageous offers. Roanoke Controls rated disadvantageous offers as less pleasant than equal offers but advantageous offers as more pleasant than equal offers. (**b**) Emotion rating across 60 rounds in MHM grouped by offer size. ABC Interventions rated equal offer as more pleasant than each disadvantageous offer as well as than each advantageous unequal offer, while ABC Controls decreased emotion rating for more disadvantageous offers but reported no difference between equal and advantageous offers. Compared with ABC Controls and Roanoke Controls, ABC Interventions rated each advantageous offer as significantly less pleasant. Every dot represents emotion rating for one individual participant in the corresponding condition. * $p < 0.05$, ** $p < 0.001$ (post hoc *t* test p-values). Error bar represents s.e.m.

Supplementary Table 1.

Demographics of original and 5th decade study sample

Variable	Interventions at Entry (N = 57)	Interventions in current study (N = 42)	Controls at Entry (N = 54)	Controls in current study (N = 36)
Mean Maternal IQ	85.4	85.5	84.5	84.1
Mean Maternal Age at Birth	19.6	18.8	20.3	20.2
Mean Maternal Early education	10.5	10.5	10.2	10
% Below Poverty	100%	100%	100%	100%
% African American	94%	98%	100%	100%

Supplementary Table 2.

Rejection rates and emotion ratings in Medium-Low-Medium conditioning type

	Rejection rate (mean ± standard error)			Emotion rating (mean ± standard error)		
	ABC Intervention	ABC Control	Roanoke Control	ABC Intervention	ABC Control	Roanoke Control
offers < 5	89.5 ± 4.8%	81.4 ± 7.6%	61.3 ± 3.9%	2.8 ± 0.4	2.6 ± 0.4	2.3 ± 0.1
offers = 5	85.9 ± 6.7%	76.8 ± 8.0%	42.4 ± 4.1%	3.1 ± 0.3	3.2 ± 0.4	3.2 ± 0.1
offers = 6	74.4 ± 8.1%	74.3 ± 8.0%	35.5 ± 3.8%	3.8 ± 0.3	3.3 ± 0.4	3.7 ± 0.1
offers = 7	55.5 ± 10.4%	68.9 ± 8.7%	16.9 ± 2.8%	4.1 ± 0.4	3.7 ± 0.3	4.5 ± 0.1
offers = 8	34.0 ± 10.4%	37.1 ± 8.7%	7.3 ± 1.9%	4.7 ± 0.4	4.7 ± 0.4	5.3 ± 0.1
offers = 9	15.3 ± 8.2%	22.2 ± 7.4%	1.6 ± 0.8%	5.3 ± 0.4	5.0 ± 0.4	5.9 ± 0.1
offers = 10	1.2 ± 1.2%	9.1 ± 5.6%	0.2 ± 0.2%	7.1 ± 0.4	7.7 ± 0.4	7.5 ± 0.1

Supplementary Table 3.

Middle life demographic indicators

Item	ABC Intervention (n = 42)	ABC Control (n = 36)	*p* value
Strength indicators			
**Early education**			
Completed high school	97.6%	75.0%	0.00
Completed college degree	28.6%	13.9%	0.09
Completed graduate degree	7.1%	2.8%	0.37
**Health**			
Overall favorable health rating	52.4%	36.1%	0.11
**Employment**			
Employed full-time	78.6%	61.1%	0.08
**Personal Resources**			
Owns home	38.1%	25.0%	0.16
Owns car	100%	97.2%	0.46
Has a checking account	90.5%	72.2%	0.04
Has a savings account	92.9%	66.7%	0.00
**Parental Relationships**			
Rates parental/parental Fig. relationships as "very close"	85.7%	58.3%	0.00
Risk indicators			
**Early education**			
Did not complete high school	2.4%	25.0%	0.00
**Health**			
Overall unfavorable health rating	9.5%	19.4%	0.18
No health insurance	16.7%	30.6%	0.12
**Employment**			
Unemployed or part-time employment	19.0%	36.1%	0.08
**Public Assistance**			
Receives Disability/SSI	2.4%	11.1%	0.14
Receives housing assistance	4.8%	8.3%	0.43
Receives food stamps	28.6%	33.3%	0.42
Receives Medicaid	14.3%	22.2%	0.27
**Civic Engagement**			
Did not vote in last presidential election	9.5%	25.0%	0.06
**Teen Parent**			
Less than age 20 at 1st child's birth	21.4%	44.4%	0.03

Supplementary Table 4.

Averaged Bayesian information criterion scores

	All participants (N =330)	ABC Intervention (N = 42)	ABC Control (N = 36)	Roanoke Control (N = 252)
Fehr-Schmidt model	28.31 ± 0.90	35.40 ± 2.62	36.53 ± 3.60	25.99 ± 0.93
Bayesian Observer model (fixed initial norm)	33.69 ± 1.09	49.63 ± 2.91	49.05 ± 3.21	28.84 ± 1.09
Bayesian Observer model (variant initial norm)	34.16 ± 1.00	45.61 ± 2.82	48.73 ± 3.21	30.17 ± 1.01
Rescorla-Wagner model (fixed initial norm)	30.76 ± 0.84	38.54 ± 2.57	39.33 ± 3.30	28.24 ± 0.84
Rescorla-Wagner model (variant initial norm)	32.06 ± 0.82	39.90 ± 2.63	40.30 ± 3.35	29.61 ± 0.80

Mean ± standard error

Supplementary Table 5.

Gender and age grouped by different types of conditioning in the ultimatum game

	MLM		MHM	
	Female	Male	Female	Male
ABC Intervention				
Sample size	6	11	14	11
Age at scan (Mean ± SD)	41.7 ± 0.6	41.2 ± 1.9	41.4 ± 1.9	40.5 ± 1.4
ABC Control				
Sample size	10	11	11	4
Age at scan (Mean ± SD)	41.4 ± 1.4	41.4 ± 1.7	41.3 ± 2.8	41.0 ± 1.4
Roanoke Control				
Sample size	77	45	80	50
Age at scan (Mean ± SD)	30.6 ± 12.9	30.4 ± 13.1	30.4 ± 12.8	28.7 ± 11.4

MLM, medium-low-medium; MHM, medium-high-medium

Supplementary Note 1

Rejection rate and Emotion rating for medium-low-medium in ultimatum game

The Group × Equality interaction in the medium-low-medium **(**MLM) type was tested by a Group × Equality analyses of covariance (ANCOVA) with Gender as the covariate. The interaction of Gender × Equality was not significant, $p = 0.423$.

See **Supplementary Fig. 1** for the illustration of rejection rate for MLM across all rounds. The Group × Equality interaction in MLM was significant, $F(2,156) = 11.037$, $p < 0.001$, $\eta^2_p = 0.124$ (**Supplementary Fig. 1b**). All three groups who underwent the MLM conditioning type had a higher rejection rate for disadvantageous offers than for equal offers (p's < 0.001). However, Roanoke Controls (28.5 ± 2.3%) had a lower rejection rate for disadvantageous offers than ABC Interventions (58.8 ± 6.2%, $p < 0.001$) and ABC Controls (59.9 ± 6.0%, $p < 0.001$), while Roanoke Controls had a lower rejection rate for equal offers (0.2 ± 0.2%) than ABC Controls (9.1 ± 5.6%, $p = 0.001$) but not lower than ABC Interventions (1.2 ± 1.2%, $p = 1.000$). The Group × Offer Size interaction in MLM was also significant, $F(6,452) = 4.326$, $p < 0.001$, $\eta^2_p = 0.053$ (**Supplementary Fig. 1c**). Specifically, Roanoke Controls rejected less offers at most of the disadvantageous offers than ABC Interventions and ABC Controls (offers < 5: Roanoke Controls < ABC Interventions, $p = 0.040$; offers = 5: Roanoke Controls < ABC Interventions, $p = 0.001$, Roanoke Controls < ABC Controls, $p = 0.005$; offers = 6: Roanoke Controls < ABC Interventions, $p = 0.003$, Roanoke Controls < ABC Controls, $p = 0.001$; offers = 7: Roanoke Controls < ABC Interventions, $p < 0.001$, Roanoke Controls < ABC Controls, $p < 0.001$; offers = 8: Roanoke Controls < ABC Interventions, $p = 0.002$, Roanoke Controls < ABC Controls, $p < 0.001$; offers = 9: Roanoke Controls <

ABC Interventions, $p = 0.022$, Roanoke Controls < ABC Controls, $p < 0.001$, see **Supplementary Table 2** for details. In the analysis for Group × Offer Size ANCOVA with Gender as the covariate, the interaction of Gender × Offer Size was not significant, $p = 0.416$.

See **Supplementary Fig. 2** for the illustration of emotion rating for MLM across all rounds. No significant Group × Equality interaction was found in MLM type, $F(2,145) = 2.023$, $p = 0.136$, $\eta^2_p = 0.027$ (**Supplementary Fig. 2a**). The main effect of equality was significant: disadvantageous unequal offers were rated as less pleasant than equal offers, $F(1,145) = 32.807$, $p < 0.001$, $\eta^2_p = 0.185$. No group difference was found, $F(2,145) = 0.338$, $p = 0.714$, $\eta^2_p = 0.005$. The interaction of Gender × Equality was significant, $F(1,145) = 6.253$, $p = 0.014$, $\eta^2_p = 0.041$. Female participants (7.9 ± 0.2) rated the equal offers as more pleasant than male participants (7.0 ± 0.2), $p = 0.005$, while no difference was found for unequal offers between female (3.9 ± 0.2) and male (3.9 ± 0.2), $p = 0.939$. A Group × Offer Size interaction in MLM was significant, $F(7,457) = 3.420$, $p = 0.002$, $\eta^2_p = 0.047$ (**Supplementary Fig. 2b**). The only group difference was that Roanoke Controls rated offers = 7 ($p = 0.034$) and offers = 9 ($p = 0.002$) as more pleasant than ABC Controls (see **Supplementary Table 2** for details). The main effect of Gender was not significant, $F(1,138) = 0.678$, $p = 0.412$, $\eta^2_p = 0.005$. The interaction of Gender × Equality was significant, $F(3,457) = 4.670$, $p = 0.040$, $\eta^2_p = 0.019$. Female participants (7.9 ± 0.2) rated the equal offers as more pleasant than male participants (7.0 ± 0.2), $p = 0.006$, while no difference was found for other offers between female and male (offer < 5, $p = 0.310$; offer = 5, $p = 0.984$; offer = 6, $p = 0.907$; offer = 7, $p = 0.952$; offer = 8, $p = 0.219$; offer = 9, $p = 0.494$).

Supplementary Note 2

Rejection rate and emotion rating for medium-high-medium in ultimatum game

The Group × Equality interaction in the medium-high-medium (MHM) type was tested by a Group × Equality analyses of covariance (ANCOVA) with Gender as the covariate. The interaction of Gender× Equality was not significant, $p = 0.417$.

Of most interest, the ABC Intervention group showed a significant Group × Offer Size interaction, $F(5,411) = 8.229$, $p < 0.001$, $\eta^2_p = 0.090$. Specifically, at each offer size corresponding to advantageous inequality, ABC Interventions rejected offers more than both ABC Controls and Roanoke Controls (offers = 11: ABC Interventions > ABC Controls, $p = 0.001$, ABC Interventions > Roanoke Controls, $p < 0.001$; offers = 12: ABC Interventions > ABC Controls, $p < 0.001$, ABC Interventions > Roanoke Controls, $p < 0.001$; offers > 12: ABC Interventions > ABC Controls, $p < 0.001$, ABC Interventions > Roanoke Controls, $p < 0.001$). Meanwhile, at almost every offer size with disadvantageous inequality, ABC Interventions and ABC Controls rejected offers more than Roanoke Controls (offers < 8: ABC Interventions > Roanoke Controls, $p = 0.003$, ABC Controls > Roanoke Controls (marginally significant), $p = 0.066$; offers = 8: ABC Interventions > Roanoke Controls, $p < 0.001$, ABC Controls > Roanoke Controls, $p = 0.026$; offers = 9: ABC Interventions > Roanoke Controls, $p = 0.002$, ABC Controls > Roanoke Controls, $p = 0.021$). All p values were Bonferroni corrected. Furthermore, ABC Interventions increased their rejection rate for more disadvantageous offers as well as for more advantageous unequal offers (i.e., rejection rates increased as a function of the inequality level). In contrast, ABC Controls only increased rejection rate for more

disadvantageous offers but not for more advantageous unequal offers. A similar pattern was found in Roanoke Controls (see **Table 2** for details). In the analysis for Group × Offer Size ANCOVA with Gender as the covariate, the interaction of Gender × Offer Size was not significant, $p = 0.372$.

See **Fig. 3** for the illustration of emotion rating for MHM across all rounds. The Group × Equality interaction in MHM was significant, $F(4,298) = 16.175$, $p < 0.001$, $\eta^2_p = 0.163$ (**Fig. 3a**). ABC Controls in the MHM conditioning type rated disadvantageous offers (4.6 ± 0.3) as less pleasant than equal offers (7.6 ±0.3), $p < 0.001$, but did not report different feelings about equal and advantageous offers (7.7 ±0.3), $p = 1.000$. Roanoke Controls in MHM type rated disadvantageous offers (4.5 ± 0.1) as less pleasant than equal offers (6.9 ± 0.1), $p < 0.001$, but advantageous offers (7.5 ± 0.1) as more pleasant than equal offers, $p = 0.003$. Importantly, the ABC Intervention group rated equal offers (8.1 ± 0.3) as more pleasant than both disadvantageous (4.9 ± 0.4), $p < 0.001$ and advantageous offers (5.9 ± 0.4), $p < 0.001$. The interaction of Gender× Equality was not significant, $p = 0.409$. A Group × Offer Size interaction in MHM was also significant, $F(6,481) = 15.322$, $p < 0.001$, $\eta^2_p = 0.163$ (**Fig. 3b**). Compared with ABC Controls and Roanoke Controls, ABC Interventions rated each advantageous offer as significantly less pleasant (offers = 11: ABC Interventions > ABC Controls, $p = 0.008$, ABC Interventions > Roanoke Controls, $p = 0.003$; offers = 12: ABC Interventions > ABC Controls, $p = 0.001$, ABC Interventions > Roanoke Controls, $p < 0.001$; offers > 12: ABC Interventions > ABC Controls, $p < 0.001$, ABC Interventions > Roanoke Controls, $p < 0.001$). Furthermore, ABC Interventions rated equal offer as more pleasant than each disadvantageous offer as well as than each advantageous unequal offer, *p's* < 0.001. On

the disadvantageous side for ABC Interventions, emotion rating decreased as the offers got more disadvantageous. On the advantageous side for ABC Interventions, equal offer was rated as more pleasant than any other offer, *p's* < 0.001, but no difference was found between the advantageous offers, *p's* = 1.000. In contrast, ABC Controls decreased emotion rating for more disadvantageous offers but reported no difference in emotion between equal and advantageous offers. For Roanoke Controls, more disadvantageous offers were rated as less pleasant while very advantageous offers (offers = 12 and offers > 12) were experienced as more pleasant than equal offers, *p's* < 0.05 (see **Table 3** for details). In the analysis for Group × Offer Size ANCOVA with Gender as the covariate, the interaction of Gender × Offer Size was not significant, $p = 0.348$.

Supplementary Note 3

Modeling results in ultimatum game

The FS model was the winning model, with the lowest average BIC across all participants and within each group of participants (see **Supplementary Table 4** for details). Thus, group comparisons on FS model-based parameters are illustrated in **Fig. 2d**. The main effect of group was found to be significant for envy, $F(2,327) = 30.367$, $p < 0.001$, $\eta^2_p = 0.156$, with both ABC Interventions (3.78 ± 0.52) and ABC Controls (3.87 ± 0.55) having a higher level of envy than Roanoke Controls (1.49 ± 0.12), *p's* < 0.001, while ABC Interventions had similar envy as the ABC Controls ($p = 1.000$). The main effect of group was also found to be significant for guilt, $F(2,326) = 39.940$, $p < 0.001$, $\eta^2_p = 0.197$, with ABC Interventions (4.12 ± 0.68) having a higher guilt than ABC Controls (1.45 ± 0.49), $p < 0.001$ and Roanoke Controls (0.47 ± 0.11), $p < 0.001$. No

difference of guilt was found between ABC Controls and Roanoke Controls, $p = 0.068$. The main effect of group was also found to be significant for inverse temperature, $F(2,326) = 14.123$, $p < 0.001$, $\eta^2_p = 0.080$, with both ABC Interventions (0.69 ± 0.05, $p = 0.001$) and ABC Controls (0.65 ± 0.06, $p < 0.001$) having a lower inverse temperature than Roanoke Controls (0.86 ± 0.02). No difference of inverse temperature was found between ABC Interventions and ABC Controls, $p = 1.000$. The main effect of Gender was not significant for envy, $F(1,326) = 0.293$, $p = 0.589$, $\eta^2_p = 0.001$, for guilt, $F(1,326) = 2.823$, $p = 0.094$, $\eta^2_p = 0.009$, or for inverse temperature, $F(1,326) = 0.798$, $p = 0.372$, $\eta^2_p = 0.002$.

Supplementary Note 4

Modeling results in multi-round trust game

The main effect of group was found significant for inverse temperature $H(2) = 8.844$, $p = 0.012$, with Roanoke Controls (0.36 ± 0.01) having a tendency to have a lower level of inverse temperature than ABC Controls (0.40 ± 0.04, $p = 0.072$) and ABC Interventions (0.42 ± 0.04, $p = 0.079$). The lower value for inverse temperature in Roanoke Controls indicated that they might be less certain about their choice preference and hence chose more randomly during the MRT than ABC Controls and ABC Interventions. No group effect was found for Inequality aversion, $H(2) = 0.990$, $p = 0.610$ (ABC Interventions, 0.76 ± 0.05; ABC Controls, 0.70 ± 0.06; Roanoke Controls, 0.76 ± 0.02), risk aversion, $H(2) = 1.026$, $p = 0.599$ (ABC Interventions, 1.30 ± 0.06; ABC Controls, 1.33 ± 0.06; Roanoke Controls, 1.26 ± 0.03), irritability, $H(2) = 0.642$, $p = 0.726$ (ABC Interventions, 0.11 ± 0.04; ABC Controls, 0.10 ± 0.04; Roanoke Controls,

0.10 ± 0.01), or irritability belief, $H(2) = 1.422$, $p = 0.491$ (ABC Interventions, 2.10 ± 0.23; ABC Controls, 2.03 ± 0.24; Roanoke Controls, 2.28 ± 0.10). All participants in each group (42 ABC Interventions, 36 ABC Controls and 252 Roanoke Controls) were included in the group comparison for each parameter except for the irritability parameter. Two participants (1 ABC Intervention and 1 Roanoke Control) were excluded from the group comparison for irritability because this parameter could not be estimated since they were not irritated during the MRT[1].

The inequality aversion parameter in the model for MRT, which quantifies how people dislike advantageous distributions, is thus analogous in definition to the "guilt" parameter in the UG. However, we did not find significant correlation between them in any group of participants (ABC Interventions, $r_s(40) = 0.189$, $p = 0.230$, ABC Controls, $r_s(34) = 0.069$, $p = 0.691$, or Roanoke Controls, $r_s(250) = -0.030$, $p = 0.633$). The reason can be that these two tasks involve very different contexts—a single interaction with several individuals in the UG vs. repeated interactions with the same individual in the MRT. Hence, their estimation based on the participants' behavior and their values may differ.

Supplementary Note 5

Midlife demographic information

Chi square analyses were used to explore group differences for midlife strength indicators and midlife risk indicators. In terms of midlife strength indicators, ABC Interventions, compared to ABC Controls, had higher "very close" relationships with their parents (85.7 vs. 58.3%, $p < 0.001$), high school attainment (97.6 vs. 75.0%, $p <$

0.001), checking accounts (90.5 vs. 72.2%, $p = 0.04$) and saving accounts (92.9 vs. 66.7%, $p < 0.001$). ABC Interventions also have the tendency to obtain higher college attainment (28.6 vs. 13.9%, $p = 0.09$) and full-time employment (78.6 vs. 61.6%, $p = 0.08$) than ABC Controls. In terms of midlife risk indicators, less ABC Interventions, compared to ABC Controls, did not complete high school (2.4 vs. 25.0%, $p < 0.001$) and had the first child before age 20 (21.4 vs. 44.4%, $p = 0.03$). ABC Controls also have the tendency to engage less in civic activity (25.0 vs. 9.5% did not vote in last presidential election, $p = 0.06$) and to be more unemployed or part-time employed (36.1 vs. 19.0%, $p < 0.001$) than ABC Interventions. The group differences were not significant for other indicators (See **Supplementary Table 3** for details).

Supplementary Note 6

Non-deception and incentivization

Participants were told that the offers in the UG task, which were actually generated by an algorithm, were proposed by different partners, but we did not specify if the partners were human or computer. We chose to generate the offers based on an algorithm because we needed to have enough advantageous offers in order to test our hypotheses. Since people don't usually give high offers (i.e., offers advantageous to the Responder) when playing as the Proposer in the UG, we would not have been able to study the reaction of participants to different levels (especially advantageous ones) of inequality if we did not control this variable. The Trustee's responses in the MRT were generated using a k-nearest neighbors sampling algorithm on known responses from real players (similar to the practice in Supplementary Reference [2]). Again, participants were

told that they, as the Investor, were playing with another partner across the whole game, and the instruction was not specific about whether the repayments were sent by another person in real-time or based on previous responses from real players or from a computer. The practice here and in the UG was aimed at providing an ecological setting for the games (social tasks) while still controlling some aspects of the game.

We provided monetary incentives (same for all groups) in both UG and MRT according to common practices used in these tasks. Participants were told that at the end of the UG one of the rounds in the UG would be randomly picked and they would be paid according to their decision on that round. In the MRT, participants were told that they would be paid based upon their earnings for the entire MRT task. In such ways, we tried to make sure different behaviors correspond to monetary differences in payments—higher gains in the games result in higher payments at the end—to motivate them to take the experiment seriously.

Supplementary References

1 Hula, A., Vilares, I., Lohrenz, T., Dayan, P. & Montague, P. R. A model of risk and mental state shifts during social interaction. *PLoS computational biology* **14**, e1005935 (2018).

2 King-Casas, B. *et al.* The rupture and repair of cooperation in borderline personality disorder. *science* **321**, 806-810 (2008).
